# Desafíos para la pertinencia cultural de fisioterapia en la atención de población mapuche en Chile

**DOI:** 10.18294/sc.2024.4849

**Published:** 2024-10-22

**Authors:** Mónica Manríquez-Hizaut, Trinidad Martínez-Campos, Nicolás Lagos-Gallardo, Jame Rebolledo-Sanhueza

**Affiliations:** 1 Magíster en Salud Pública. Profesora asistente, Departamento de Kinesiología, Facultad de Medicina, Universidad de Chile, Santiago, Chile. mmanriquezh@uchile.cl Universidad de Chile Departamento de Kinesiología Facultad de Medicina Universidad de Chile Santiago Chile mmanriquezh@uchile.cl; 2 Licenciada en Kinesiología. Escuela de Kinesiología, Facultad de Medicina, Universidad de Chile, Santiago, Chile. trinidadmartinez@ug.uchile.cl Universidad de Chile Escuela de Kinesiología Facultad de Medicina Universidad de Chile Santiago Chile trinidadmartinez@ug.uchile.cl; 3 Licenciado en Kinesiología. Escuela de Kinesiología, Facultad de Medicina, Universidad de Chile, Santiago, Chile. nicolaslagos@ug.uchile.cl Universidad de Chile Escuela de Kinesiología Facultad de Medicina Universidad de Chile Santiago Chile nicolaslagos@ug.uchile.cl; 4 Autora de correspondencia. Magíster en Psicología Comunitaria. Profesora asistente, Departamento de Kinesiología, Facultad de Medicina, Universidad de Chile, Santiago, Chile. jame.rebolledo@uchile.cl Universidad de Chile Departamento de Kinesiología Facultad de Medicina Universidad de Chile Santiago Chile jame.rebolledo@uchile.cl

## Abstract

La rehabilitación en fisioterapia debe atender a las preferencias culturales de la población y, como principal herramienta, la actividad física debe analizarse desde un enfoque intercultural en salud. Este estudio busca caracterizar las experiencias de atención kinésica/fisioterapéutica relacionadas con la actividad física desde una visión intercultural. A partir de un abordaje cualitativo, entre junio de 2021 y marzo de 2023, se realizaron ocho entrevistas individuales semiestructuradas a personas adultas mapuches, profesionales del área y trabajadoras del programa de salud indígena de centros de atención primaria de una comuna urbana de Santiago de Chile. Existen desafíos para la implementación de la interculturalidad, como el distanciamiento entre programas de salud, falta de formación profesional y la discriminación hacia la comunidad mapuche. En las prestaciones de rehabilitación, en particular en las prácticas de actividad física como estrategia de tratamiento hacia personas mapuches, la pertinencia cultural es escasa o inexistente.

## INTRODUCCIÓN

El concepto de salud tiene una definición amplia que va desde un enfoque biomédico hasta una dimensión cultural. La Organización Mundial de la Salud (OMS) define salud como “un estado de completo bienestar físico, mental y social, y no solamente la ausencia de afecciones o enfermedades”^1^. Aun cuando el estado de completo bienestar puede ser considerado irreal, esta comprensión ha permitido entender la salud y enfermedad como un continuo y un estado de equilibrio permanente que incluye diversos factores biológicos y sociales en continua interacción y producción^2^. Desde una perspectiva cultural, se comprende que todos los pueblos desarrollan diversos modelos de salud para abordar las enfermedades y mantener o recuperar la salud^3^. 

La salud colectiva aborda el continuo salud-enfermedad desde una perspectiva histórica y situada, que da cuenta de cómo los grupos sociales organizan los servicios de salud, incluyendo la conjugación de distintos saberes^4^, entre ellos, las visiones sobre salud de distintas culturas indígenas. Menéndez^5^ plantea que a través de los procesos de salud-enfermedad se plasman la mayoría de los conflictos ideológico-culturales y se visualizan con mayor transparencia y menos sesgos los conflictos sociales que muchas veces son analizados desde otras perspectivas del conocimiento. Es decir, al observar las dinámicas existentes en los fenómenos de salud-enfermedad, también estamos indagando en las asimetrías de poder presentes en la sociedad como, por ejemplo, las inequidades étnicas o de género. Por tanto, es relevante comprender los modos en que se desarrolla la interculturalidad en salud, la cual es considerada como un marco de acción que busca promover la igualdad de trato entre los distintos grupos culturales, considerando la salud como prioridad y derecho fundamental. Además, implica la capacidad de los profesionales de la salud de integrar los conocimientos con las creencias y las prácticas tradicionales al momento de enfrentar una situación de salud-enfermedad^6^. 

La Organización Panamericana de la Salud ha establecido la interculturalidad en salud como prioridad para los pueblos indígenas de Latinoamérica, promoviendo el desarrollo de políticas sanitarias orientadas por los principios de atención integral, autodeterminación de los pueblos, revitalización cultural y reciprocidad de las relaciones^7^. En Chile, se reconoce la interculturalidad en salud como un principio transversal al sistema de salud, especialmente, en la atención primaria de salud (APS), la cual es gestionada localmente desde los enfoques territoriales y de salud familiar y comunitaria^8^. En este contexto, se esperaría mayor apertura al diálogo intercultural; sin embargo, y a pesar de la valoración positiva que profesionales de APS puedan tener de la salud indígena, reconocen asimetrías entre los sistemas de salud e incertidumbres respecto a su complementariedad^9^.

En la actualidad, el Estado chileno reconoce diez pueblos indígenas: mapuche, aymara, rapa nui, atacameño, quechua, colla, diaguita, kawashkar o alacalufe, yámana o yagán y selk’nam, que representan el 12,8% de la población nacional, lo que equivale a poco más de dos millones de personas. La población mapuche es la más numerosa, con un 79,84% y reside principalmente en las regiones de la Araucanía y Metropolitana^10^. La población indígena presenta un alto grado de desigualdad, que se expresa en mayores tasas de mortalidad general que la población no indígena^11^. En relación con la situación económica, la población indígena tiene una mayor incidencia de pobreza (8,8%) y pobreza extrema por ingresos (2,7%) en comparación con la población no indígena (6,2% y 1,9% respectivamente)^12^. Y desigualdades en la calidad del empleo y en educación, por ejemplo, el promedio de escolaridad de la población indígena es 10,3 años, mientras que la población no indígena alcanza en promedio 11,3 años de estudios. Adicionalmente, las personas indígenas presentan mayor afiliación al sistema de salud público (86,4%) en comparación a la población no indígena (77,2%)^13^. 

La salud mapuche tiene una cosmovisión sobre la vida y el bienestar que considera tanto los orígenes ancestrales y la espiritualidad, como el equilibrio con la naturaleza, por tanto, la ruptura de este equilibrio se manifestaría como enfermedad^14^. Este modelo mapuche de salud-enfermedad se desarrolla a través de la práctica de agentes tradicionales, quienes no siguen una formación estandarizada como la del modelo biomédico, sino que adquieren un don, por medio de sueños, visiones en una conexión íntima con la naturaleza y los antepasados^15^. 

Dada esta especificidad cultural, en ocasiones, las personas indígenas desconfían de la medicina occidental, lo cual las excluye de programas de promoción de salud. Por otro lado, la desconexión con las prácticas tradicionales ha demostrado llevar a una peor condición física, mental y de bienestar general^16^. Adicionalmente, la estigmatización y discriminación de las personas mapuche se acentúa en el contexto sanitario, donde se reporta que el 82% de las personas adultas mapuche se sienten despreciadas y tratadas como inferiores dentro de los servicios de salud^17^.

Con el objetivo de reducir la brecha entre los sistemas de salud indígenas y occidentales, con base en el mutuo respeto y el igual reconocimiento de sus sistemas de conocimiento, se propone la salud intercultural como un conjunto de políticas y acciones para conocer e incorporar la cultura de las personas en el proceso de atención^18^, a través del reconocimiento de creencias sobre salud para avanzar en el diálogo de saberes^19^. El enfoque de interculturalidad busca lograr la equidad en salud, entendida por organismos internacionales como la “lucha por la imparcialidad y la justicia mediante la eliminación de las diferencias que son innecesarias y evitables”^20^. No obstante, estudios comparados en la región latinoamericana^21^ señalan que los intentos de articulación de enfoques culturales dentro del sistema de salud no han sido efectivos, evidenciando racismo institucionalizado en diversos sectores del sistema de atención de salud. 

En Chile, el Ministerio de Salud reconoce la interculturalidad en salud como acciones desarrolladas entre equipos de salud y facilitadores interculturales para “cubrir las necesidades de accesibilidad, oportunidad en la atención de morbilidad, adecuación de la organización de salud y atención de salud apropiada a las necesidades de los pueblos”^22^. El primer programa de salud intercultural nace en el año 1992, cuyos ejes centrales fueron la capacitación, la investigación, el conocimiento comunitario y la docencia. En el año 2008, se crea el Programa Especial de Salud y Pueblos Indígenas (PESPI), el cual buscó consolidar un sistema de salud intercultural que reconociera que los sistemas médicos son limitados para resolver los problemas actuales de salud, admitiendo la existencia y validez de otros sistemas de curación, como los indígenas. Este consta de tres ejes principales: equidad, enfoque intercultural y participación social indígena^23^. Distintos estudios han evaluado esta política caracterizando su implementación como un espacio complejo, heterogéneo y con múltiples tensiones^24^^,^^25^^,^^26^^,^^27^. Sin embargo, no existen estudios nacionales específicos sobre la interculturalidad en el ámbito de la rehabilitación o de la kinesiología-fisioterapia.

El deporte y la actividad física son prácticas valoradas en el ámbito de la kinesiología-fisioterapia y reconocida por sus beneficios para la salud^28^^,^^29^^,^^30^. Para las culturas indígenas de Centroamérica y América del Sur estas actividades corporales transmiten los mitos y valores culturales que congregan el mundo material e inmaterial de cada pueblo^31^. Se puede considerar la actividad física y el deporte como conceptos polisémicos y culturales, históricamente situados y, por tanto, relevantes al momento de pensar la rehabilitación desde un enfoque intercultural. Un estudio con mujeres maoríes demuestra que la consideración de las preferencias étnicas como la modificación del entorno para la realización de distintas actividades permitió aumentar su participación y cumplimiento de pautas de actividad física^32^. 

Para la kinesiología-fisioterapia, disciplina que se centra en el movimiento humano y su relación con el medio externo, el deporte y la actividad física toman relevancia y protagonismo en las atenciones de salud. Además, los programas de rehabilitación que se desarrollan en la atención primaria del sistema público de salud chileno deberían trabajar desde un enfoque intercultural, lo cual no se ha indagado hasta la fecha. Ampliar el conocimiento respecto a la pertinencia cultural de las prácticas de los servicios de kinesiología-fisioterapia contribuirá a mejorar la integralidad de la atención y su pertinencia cultural. El objetivo de este estudio fue caracterizar las experiencias de atención kinésicas-fisioterapéuticas relacionadas con la actividad física, de personas adultas mapuche, profesionales del área y trabajadoras del programa de salud indígena de centros de atención primaria de una comuna urbana de Santiago de Chile, desde una visión intercultural. 

## METODOLOGÍA

Se realizó un estudio cualitativo^33^ sobre la pertinencia cultural en la atención fisioterapéutica a personas mapuche en una localidad urbana de Santiago de Chile. Cabe aclarar que en esta investigación se usa la palabra “mapuche” tanto para el singular como para el plural, dado que la palabra en mapudungun contiene el plural “mapu” tierra y “che” gente. 

Las personas participantes incluidas en este estudio fueron profesionales de kinesiología-fisioterapia que se desempeñan en programas de atención primaria de salud (APS), personas mapuche usuarias de los servicios de APS y agentes claves del programa PESPI, participantes de su implementación comunal. Consideramos un perfil para cada agente clave, según los siguientes criterios de inclusión: profesionales de kinesiología en servicios de APS con al menos un año de experiencia, con desempeño en el área de musculoesquelético y/o respiratorio. El grupo de agentes claves del programa PESPI debían ser personas que tuvieran roles de dirección, gestión y/o liderazgo en áreas de interculturalidad y pertinencia cultural dentro del servicio de APS. Finalmente, las personas usuarias debían identificarse como pertenecientes al pueblo mapuche y haber recibido atención kinesiológica en APS en los últimos dos años. A partir de cada perfil se fue reclutando a las personas participantes del estudio. El número final de participantes fue de ocho personas (Tabla 1), definido por un muestreo teórico, según la información producida en las entrevistas, con el propósito de identificar temas y experiencias a profundizar, que guiaron el reclutamiento de participantes. Esta composición final cumplió con criterios de saturación, entendido como el momento en que el equipo investigador disponga de elementos necesarios para construir una teoría comprensiva, novedosa, problematizadora y convincente sobre el tema^34^. 

**Tabla 1:** Caracterización sociodemográfica de las personas participantes. Santiago, junio de 2021 y marzo de 2023.

Variables sociodemográficas	Personas usuarias mapuches	Profesionales de kinesiología-fisioterapia	Agentes claves del Programa Especial de Salud y Pueblos Indígenas (PESPI)
**Sexo**			
Varón	1	1	0
Mujer	2	2	2
**Edad (promedio de años)**	61	34	45
**Ascendencia indígena**			
Sí	3	0	1
No	0	3	1
**Años de experiencia laboral en la comuna (promedio)**	No aplica	7	10

Fuente: Elaboración propia.

La convocatoria se realizó a través de la estrategia de portero, en la cual personas que tenían el contacto del perfil de participantes que buscábamos mediaron la invitación y, posteriormente, el equipo de investigación formalizó la invitación y realizó el proceso de consentimiento informado. De las personas invitadas a participar, solo una se negó por no tener disponibilidad de tiempo. 

La producción de datos se realizó mediante entrevistas cualitativas semiestructuradas individuales, conducidas por una dupla del equipo de investigación, compuesta por un/a estudiante de Kinesiología de tercer año y una tutora (académica) del departamento de Kinesiología. Previo al inicio del trabajo de campo, se realizó una entrevista piloto a modo de capacitación para parte del equipo de investigación en formación y, además, con el fin de poner a prueba la pauta de preguntas.

Las entrevistas fueron guiadas por una pauta de preguntas abiertas y flexibles, orientadoras del proceso de producción de la información expresada en las respuestas verbales y no verbales de la persona entrevistada^35^. La guía de entrevista comenzaba con una serie de preguntas de identificación de las personas, su relación con el pueblo mapuche y sus creencias en torno al proceso de salud y enfermedad. Luego se indagaba sobre prácticas de abordaje o ajustes de las preferencias culturales en la atención y sus consideraciones dentro de la actividad física. Finalmente, se consultaba sobre la percepción de la implementación de la interculturalidad en la APS, y un espacio para otros comentarios, dudas e inquietudes. La guía fue analizada luego de cada entrevista, introduciendo cambios que servían de apoyo para el/a investigador/a en formación. El total de entrevistas fueron realizadas entre junio de 2021 y marzo de 2023, para cerrar el muestreo teórico. La totalidad de entrevistas se realizó por videollamadas mediante una cuenta institucional de Zoom y tuvieron una extensión de entre sesenta y noventa minutos. Estas sesiones fueron grabadas en audio y luego transcritas literalmente. 

Se realizó análisis de contenido cualitativo, pues permite interpretar en profundidad los datos recogidos y explicar fenómenos sociales complejos^36^. Esta orientación metodológica brinda procedimientos sistemáticos para la descripción del contenido de los datos, teniendo como propósito inferir conocimientos relativos a sus condiciones de producción. Cada entrevista fue analizada por dos investigadores independientes, mediante estrategia de codificación^36^. Se realizó un análisis deductivo guiado por los temas principales de las entrevistas, y luego inductivo considerando las temáticas emergentes. El procedimiento consistió en dar una denominación común a un conjunto de datos que compartían una misma idea, para descubrir relaciones y así comenzar a codificar, generando unidades condensadas de significado, que fueron etiquetadas, formando códigos. Luego fueron agrupados en categorías sobre la base de sus semejanzas. Finalmente, las propiedades que se incluyeron dentro de una categoría se etiquetaron y se dividieron en dimensiones para ser analizadas^36^. El equipo en conjunto definió las dimensiones que representaban los principales resultados del estudio. La gestión de los datos, así como el proceso de codificación fue realizada con apoyo del software de análisis cualitativo ATLAS.TI.

Este estudio fue aprobado por el Comité de Ética de Investigación en Seres Humanos de la Facultad de Medicina de la Universidad de Chile (acta No. 008/10, de mayo de 2022), todas las personas participantes dieron su consentimiento. Además, el equipo de investigación realizó una jornada de devolución de los resultados del estudio con personas trabajadoras del programa PESPI y las autoridades de la comuna, con el fin de exponer los hallazgos de esta investigación, de manera que sea un aporte a las perspectivas de atención en salud con un enfoque intercultural.

## RESULTADOS

Los hallazgos de este estudio se organizan en dimensiones que explican la falta de implementación de la interculturalidad en los servicios de rehabilitación en el contexto de atención primaria en una comuna urbana. Comenzamos con una presentación del caso y las relaciones relevantes para el fenómeno de estudio, considerando el contexto de organización del sistema de salud local. Posteriormente, nos referimos a las percepciones sobre la interculturalidad en la práctica de actividad física y sobre el sistema de salud y la atención kinésica o fisioterapéutica específicamente.

### Caracterización del caso de estudio

El caso estudiado se sitúa en una comuna urbana de la Región Metropolitana, específicamente en las actividades implementadas entre programas municipales de una dirección de salud comunal. Los profesionales de kinesiología-fisioterapia se desempeñan en el ámbito clínico, particularmente, en el área de rehabilitación y actividad física. Con relación a las agentes claves del Programa Especial de Salud y Pueblos Indígenas (PESPI), una de ellas cumple la función de coordinar la implementación del Programa, y la otra se desempeña como facilitadora intercultural, además de ser presidenta de una organización mapuche de la comuna. Por último, las personas usuarias entrevistadas se identificaron como descendientes mapuche, que recibieron atención kinésica en alguno de los Centros de Atención Primaria de la Comuna entre los años 2021 y 2022. Una de estas personas estaba vinculada además al PESPI.

Los programas de rehabilitación y promoción de salud no tienen una orientación específica al trabajo con población indígena; no obstante, se desarrollan en el marco de la atención primaria de salud con un modelo de atención integral y comunitario, el cual incluye la interculturalidad como enfoque de acción. Los profesionales de estos programas, además de no ser descendientes directos del pueblo mapuche, refieren no tener experiencias personales con sus prácticas culturales. 

El programa PESPI desarrolla actividades de salud mapuche, fortalecimiento de organizaciones indígenas en la comuna y capacitaciones a público general y a profesionales de salud. La encargada del programa tampoco tiene experiencias personales cercanas; sin embargo, debido a su rol se ha familiarizado con las prácticas de salud mapuche y al trabajo de las organizaciones indígenas en el territorio.

Por su parte, quien se desempeña como facilitadora intercultural, trabaja esporádicamente en el programa PESPI, y nació y creció en su familia mapuche según las tradiciones de su pueblo. Su primera lengua fue el Mapudungun, que utiliza hasta el día de hoy. Además de los conocimientos de la lengua, ella atribuye parte importante de su trabajo a su experiencia de vida en el entorno y prácticas de su pueblo:

“*Soy nacida y criada en el campo y afortunadamente nací a la orilla del fogón y tengo todas las atenciones como en nuestra cultura mapuche y el conocimiento de mi primera lengua materna el mapudungun que manejo actualmente muy bien. Tener todos esos contactos con la tierra, de toda la naturaleza, los animales, las aves, el río, el cerro y las siembras, todas esas maravillas, es que hoy en día entrego ese conocimiento*”. (Facilitadora intercultural)

En el caso de las personas usuarias, a pesar de que algunas de ellas habían nacido en zonas rurales, en el seno de familias con costumbres del pueblo mapuche, estas se iban perdiendo a medida que fallecían las personas mayores y las generaciones jóvenes migraban hacia las ciudades, razón a la que le atribuían una importante desconexión con las prácticas culturales, lo cual valoraban negativamente:

“*Me siento perteneciente al pueblo mapuche desde la manera en que yo me perdí, creo que a mucha gente le pasa eso, porque es tanta la desvinculación. Yo soy de la Warria, o sea le llamamos Warria a la ciudad* [...] *hay cosas que se nos salen de las manos, malas decisiones, muchas veces nosotros nos sentimos como desconectados, desconectados de la tierra*”. (Persona usuaria mapuche)

Dos personas usuarias relataron que, en su vida en la ciudad, habían tenido la necesidad de reencontrarse con las prácticas de su pueblo, ya sea retornando a las comunidades donde nacieron o acercándose a organizaciones indígenas que buscaban rescatar dichas prácticas y que, en el caso de este estudio, eran parte del programa PESPI, que había desarrollado prácticas de salud mapuche desde las propias organizaciones.

El trabajo de las organizaciones mapuche en el territorio urbano era considerado clave para que las personas puedan reencontrarse con las prácticas de su pueblo, y desarrollarlas en conexión con su actual territorio. Para la facilitadora intercultural este encuentro con la organización mapuche en el territorio urbano fue clave para valorar su propia sabiduría y reconocer los saberes y prácticas culturales:

“...*fui fortaleciendo mi kimün, nuestra sabiduría, y poder encontrarme con un grupo de mis propios hermanos de mi pueblo y que empezaron a valorar… usted sabe mucho, usted es muy especial, usted sabe hacer sopaipillas, preparar mate, entonces eso me empezó a tirar para arriba* [animar]” (Facilitadora intercultural)

### Percepción sobre interculturalidad en actividad física

La percepción sobre la necesidad de incluir interculturalidad en las prácticas de actividad física está mediada por la comprensión de lo que implica ser mapuche en la ciudad, por prácticas de ocultamiento y discriminación étnica, y por la falta de formación y cuestionamiento por parte de los profesionales participantes del caso. Adicionalmente, emergen las posibles malinterpretaciones de la interculturalidad en el actual contexto de la práctica de actividad física en la atención kinesiológica-fisioterapéutica. A continuación, se abordan estos resultados.

#### Ser champurria: Comprensión de “ser mapuche urbano”

Las personas plantearon en sus relatos la existencia de dos tipos de personas mapuche. La primera se fundamentaba en la persistencia de costumbres y tradiciones y ser originario de la zona sur, lo que se reflejaría en el uso de la lengua mapudungun o ser descendiente de dos progenitores mapuche, lo que era caracterizado como mapuche “real”. La segunda sería lo que denominaban como “champurria”, ya sea producto del mestizaje o bien por haber nacido en la ciudad, y no crecer en contacto con las prácticas culturales mapuche. Esta distinción fue identificada por los profesionales de rehabilitación para justificar formas de relacionarse diferentes:

“*Hay gente que son de origen mapuche, pero están más acostumbradas con la sociedad actual a diferencia de otras personas que siguen sus ritos, su cultura, entonces ahí la relación es con dos personas distintas*” (Profesional de kinesiología-fisioterapia)

“*Nosotros no hemos tenido usuarios mapuche, mapuche, mapuche, que hablen solamente mapudungun… sino que son personas que son mapuche, que saben el español y se comunican en español*”. (Profesional de kinesiología-fisioterapia)

Las citas muestran la idea de dos categorías de personas mapuche con diferente aproximación a su cultura. Esta diferencia es también reconocida por la facilitadora intercultural y las personas usuarias.

“*Aquí hay personas mapuche, pero urbanas, que no manejaban muchas cosas pero tenían todas las ganas*”. (Facilitadora intercultural)

“*Yo soy champurria, nacido y criado en Santiago, tengo otro concepto de vida digamos, yo soy más winka que mapuche*”. (Persona usuaria mapuche)

Esta distinción tendrá implicancias en las prácticas de salud, pues para los profesionales no es evidente la necesidad de ajustes, y para las personas usuarias, familiarizadas con el sistema de salud occidental y urbano no habrá necesidad de adecuaciones culturales en las atenciones respecto a la actividad física.

#### Ocultamiento por experiencias de discriminación

Por parte de los tres grupos de participantes del caso, se identificó una práctica común de ocultamiento, que algunos entendían como timidez de la población mapuche. No obstante, a partir de la historia de vida de la facilitadora intercultural, estas prácticas se podrían interpretar como estrategias de ocultamiento para evitar la discriminación o bien como producto de la discriminación.

Para las y los profesionales de kinesiología-fisioterapia las personas mapuche tendían a ser más discretas al momento de expresar sus ideas o participar en instancias de actividades grupales que se realizaban en centros de atención primaria. No explicitaban espontáneamente su pertenencia étnica y evitaban dar sus apellidos mapuche, buscando prevenir comentarios negativos.

“*Son más bien personas más tímidas, más recatadas, que no siempre lo van a decir a viva voz, o incluso a uno mismo… uno se entera muy de pasada, por así decirlo, de que la persona es mapuche porque no es algo que lo va a verbalizar en un taller, por ejemplo* [...] *tratan de hacerlo pasar muy desapercibido para que no existan diferencias con ellos, para que no los vayan a agredir, porque siempre están como a la defensiva*”. (Profesional de kinesiología-fisioterapia)

El sistema escolar chileno era identificado como una de las instituciones en las que se había generado rechazo hacia la cultura mapuche y prácticas de discriminación, debido a la imposición hegemónica de la cultura occidental imperante en Chile, que en concreto implicaba la prohibición del uso de la lengua mapudungun y la discriminación étnica por medio de burlas con relación a la fisionomía, experiencia que vivió tanto la facilitadora intercultural como sus hijas, y que tuvieron como consecuencia sentir que ser mapuche era un problema, del cual debía alejarse.

“*Pasé una discriminación muy grande por ser mapuche o por ser mujer, de todo, porque tenía la primera lengua que me dio alma mapuche, me costó un poco igual, a cualquiera le cuesta tomar otro idioma y poder hablarlo bien… la escuela me trató muy mal, primero porque me quitó la lengua que yo tenía original, que es mi vida, es mi forma de vivir, entonces eso me marcó mucho en mi vida y por harto rato yo sufrí de esa discriminación, de dolores en mi corazón, discriminación por ser mapuche, porque su pelo es así, porque su cara es así* […] *yo no quería saber nada, ni siquiera de mi apellido porque estaba muy dolida*”. (Facilitadora intercultural)

También las personas usuarias referían experiencias de discriminación o sentirse avergonzadas y, por tanto, reconocían ocultamiento y aversión a sus apellidos mapuche, como símbolo de identificación, aun cuando con sus propias experiencias de búsqueda de sus raíces étnicas consideraban que era algo que iba quedando en el pasado.

“*El mapuche siempre tuvo vergüenza al chileno, a su forma de hablar a su forma de caminar que se yo, entonces eran muy tímido, pero ahora, ya eso se ha pasado*” (Persona usuaria mapuche)

Cuestiones como esta producían que las personas mapuche quedaran invisibilizadas y marginadas de las prácticas colectivas que se realizaban en APS, incluso que evitaran participar de este tipo de actividades. Aun siendo parte, no sentían que podían compartir sus preferencias, lo que dificultaba la implementación de instancias interculturales que les permitieran tanto a las personas mapuche como a las no mapuche reconocer y valorar la cosmovisión indígena y con ello superar las prácticas de ocultamiento. Esta valoración colectiva fue clave para la facilitadora intercultural, permitiéndole el empoderamiento y reencuentro con la propia cultura, y sus formas de expresión y opinión, lo que posibilitaba una participación íntegra y completa en las distintas áreas de la atención primaria para el logro de la implementación de la salud intercultural.

“*Hoy en día estoy bien parada, estoy floreciendo, estoy dando frutos de mi vida como mapuche, de no avergonzarme de mi vestimenta y ojalá todos me puedan mirar y darse vuelta a mirarme por mi vestimenta que les llama la atención y no como antiguamente cuando yo me vestía*”. (Facilitadora intercultural)

#### Falta de cuestionamiento y formación profesional

Al conversar con los profesionales de rehabilitación sobre la necesidad de ajustes de las prácticas de atención y de actividad física desde una perspectiva intercultural, se tornaba un tema difícil de abordar por no tener experiencias al respecto. Aunque algunos lograban identificar diferencias en las personas usuarias, tanto en la realización de ejercicios físicos específicos y con fines recreativos, como con el trabajo en grupos, no reparaban en una necesidad de indagar sobre sus preferencias. Por otro lado, señalaban que las únicas adaptaciones se relacionaban con la condición biológica de las personas, pues respondían a criterios profesionales dentro de las prácticas de salud occidental.

“*Cuesta agarrarlos, cuesta que participen o incentivarlos, no están acostumbrados al ejercicio de manera recreativa, sino que están más adaptados al ejercicio funcional como dándole alimento a los animales, cuesta agarrarlo por ese lado de entretención, pero sí son activos*”. (Profesional de kinesiología-fisioterapia)

“*Se adapta dependiendo de la condición física de la persona, pero no hay diferenciación* [...] *yo no me atrevería a decir una separación por pueblo originario, sino más bien con la situación que la persona se encuentre, o sea, si una persona está con artrosis hay ciertos ejercicios que no puede hacer, en otro contexto es solamente adaptación dependiendo de la condición biológica que se encuentre la persona*”. (Profesional de kinesiología-fisioterapia)

Por otro lado, incluso cuando las y los profesionales podían pensar en formas de incluir acciones de sanación durante la terapia, reconocían que no existían instancias para facilitar estas solicitudes.

“*No me ha tocado que me pidan hacer alguna finalización de sus terapias de manera distinta. Lo veo difícil, si me lo quisieran comunicar casi siempre están en actividades grupales, porque tratamos de pasar a taller rápidamente*”. (Profesional de kinesiología-fisioterapia)

Finalmente, durante la conversación, las y los profesionales asumían como posible introducir ajustes que permitieran una atención pertinente, algunos cuestionaban más la pertinencia biomédica o clínica de dichas intervenciones, otros se referían más a comprender los procesos de conciencia de enfermedad y a utilizar recursos de la cosmovisión mapuche, por ejemplo, se valoraba el “autoconocimiento” que lograban las personas para estar atentos a su cuerpo y prevenir enfermedades. Por otro lado, se reconocía una falta de conocimientos sobre cómo implementar la interculturalidad, y tenían la percepción de que no se realizaban actividades diferenciadas para no discriminar. Un participante aludía a que la misma atención implicaba la misma calidad, por lo tanto, cualquier ajuste solo sería necesario en el ámbito clínico por condición física. Otra participante, percibía distinciones a favor y distinciones negativas, lo que evidenciaba que, ante la idea de igualdad como homogeneidad, se podrían transgredir o al menos no considerar costumbres culturalmente distintas.

“*Solamente se adapta la atención dependiendo de la condición física de la persona, pero no hay diferenciación… creo que en general a todas las personas hay que atenderlas igual, con la misma pasión y con la misma calidad*”. (Profesional de kinesiología-fisioterapia)

“*Podríamos hacer distinciones, pero en favor, nunca una distinción desde la vereda negativa, sino que, a lo mejor si quieres una atención distinta y como nosotros no estamos en conocimiento, a lo mejor si necesitamos ese espacio. Y nosotros en esta parada de que todos somos iguales, y que todos tenemos que participar por igual, estamos pasando a llevar y transgrediendo algo que es parte de sus ritos y de sus costumbres, entonces, en ese sentido sería súper enriquecedor aprender y conocer y poder mejorar*”. (Profesional de kinesiología-fisioterapia)

#### Malinterpretación y folklorización a partir de la interculturalidad

Las personas participantes con mayor vinculación al pueblo mapuche, es decir que conocían sobre su cosmovisión, reconocían diferencias en las prácticas y usos del ejercicio físico. No se reconocían como prácticas vinculadas a salud o como formas de tratamientos, más bien eran prácticas de actividad física cotidianas, y asociadas a instancias ceremoniales, vinculadas a la vida en comunidad.

“*Entonces el pueblo mapuche no tiene eso de movilidad, mover el brazo, mueva las piernas, haga esto, nosotros no tenemos esa cultura*”. (Persona usuaria mapuche)

“[No es] *hacer el ejercicio porque me quiero sentir bien, o porque mi cuerpo necesita tener un ejercicio, el pueblo mapuche no lo vemos de esa manera, si no que todos los ejercicios que ocurren es a través de la ceremonia* [...] *un juego de chueca o el palin que lo llamamos nosotros, compartir un momento de jugar, es ceremonia para nosotros, entonces nosotros acompañamos con la música; la trutruca, uno está todo el rato tocando el kultrún, es una forma de estar haciendo el ejercicio, pero como contexto mapuche, no lo hacemos por decirle un ejemplo porque quiero bajar de peso*”. (Facilitadora intercultural)

La práctica del juego o el ejercicio físico estaban inmersos en un contexto de práctica cultural, de cuidado comunitario y desarrollo espiritual, más que de cuidado individual. Lo que no se condecía con las formas de entendimiento del ejercicio que posee la cultura occidental y los propios profesionales de kinesiología-fisioterapia. Un usuario, buscando articular ambas prácticas, proponía usar implementos de los juegos tradicionales mapuche como elementos de entrenamiento en los “movimientos kinesiológicos” del tratamiento de rehabilitación:

“*Toman el palín, ¿Conoce esto? el palín, ya toma el palín, muévalo, movimientos de caderas, muévelo pa allá el palín, muévelo pa allá el palín, ¿te das cuenta o no?, lo pueden hacer, hay muchos movimientos que son kinesiológicos, que lo pueden usar como herramientas del pueblo mapuche*”. (Persona usuaria mapuche)

La facilitadora intercultural era enfática en señalar que la pertinencia cultural no se hacía efectiva con la sola instrumentalización del ejercicio con implementos propios de las tradiciones mapuche, pues no necesariamente seguían las lógicas y los fines de la cultura occidental, sino que eran un medio para la transmisión de ritos culturales y ceremoniales. Por lo tanto, se estarían incorporando estas herramientas sin un sentido cultural, transgrediendo los principios de respeto y valoración que proponía el enfoque intercultural.

“*Nuestra danza u otro juego es descalzo* [...] *tiene una razón porque nuestra pisada con la tierra es fundamental, nuestra conexión, el pie debe tener ese contacto con la tierra. Por eso nosotros muchas veces las danzas las hacemos en la tierra, no en el cemento, a mí me piden una ceremonia en un cemento o departamento o en un lugar donde no es apto yo no lo hago porque realmente ahí yo estoy folklorizando o vendiendo mi cultura*”. (Facilitadora intercultural)

### Percepción sobre el sistema de salud y la atención kinesiológica/fisioterapéutica

Otros factores que explicaban la falta de interculturalidad en las atenciones kinésicas tenían relación con la falta de comunicación y colaboración entre el programa de salud indígena (PESPI) y los demás programas de salud. Por otro lado, desde el desconocimiento se visualizaban los sistemas de salud mapuche y occidental chileno, como sistemas paralelos desde una diferencia que más bien hablaba de una coexistencia de diversas prácticas culturales en lugar de un diálogo intercultural.

#### Distancia entre el Programa Especial de Salud y Pueblos Indígenas y los otros programas de salud

Existe escaso conocimiento sobre el programa PESPI por parte de las y los profesionales de kinesiología-fisioterapia. Reconocían que hay una falta de información y de divulgación, así como falta de prioridad desde los cargos directivos, pues a pesar de trabajar en atención primaria en un mismo territorio, no conocían más allá de los nombres de las personas encargadas.

“*No conozco el programa PESPI* [...] *te muestra que falta información, falta divulgación, porque llevo 3 años trabajando y he escuchado dos o tres veces del programa*”. (Profesional de kinesiología-fisioterapia)

No obstante, los profesionales reconocían la necesidad de contar con herramientas que les permitieran comunicarse adecuadamente y acercarse a la cultura de otras personas, incluidos otros pueblos indígenas y personas extranjeras.

“*Como profesional de salud deberíamos estar preparados para cualquier situación, de hecho, incluye poder atender a una persona de origen haitiano con una buena comunicación, con una persona mapuche, diaguita, aymara, que exista la comunicación fluida*” (Profesional de kinesiología-fisioterapia)

Por parte del programa PESPI, se reconoce que existen dificultades para la participación de funcionarios/as en capacitaciones y talleres sobre salud intercultural, debido a la falta de tiempo para destinar a estas actividades por sobre las de su propio trabajo o su horario laboral.

“…*yo creo que esto tiene que venir más de arriba, nosotros hacemos todo el esfuerzo, pero sigo diciendo que es una pincelada, muy pequeñito, porque sí, mi deseo es entregarle la capacitación a todos los profesionales del CESFAM* [Centro de Salud Familiar], *pero no es así, ellos quisieran pero no están por cumplir su horario de trabajo* [...] *van a ir unos 5, no van a ir los 30 o 50 que deberían estar, no se puede*”. (Facilitadora intercultural)

Por otro lado, para la encargada comunal del PESPI, no solo sería un problema de disponibilidad de horario, sino de voluntad y directriz política desde el proyecto comunal y la dirección de salud en su totalidad. Para que existiera diálogo intercultural entre profesionales de la salud se debía obtener la aprobación y el interés de la jefatura o de la dirección de salud.

“*Si la persona en este caso el director o directora de salud o los alcaldes de la comuna, no le interesa esto, ni más que yo le de 24/7, no va a funcionar*”. (Encargada Comunal)

El trabajo del PESPI en este estudio se centró en fortalecer el acompañamiento a personas mapuche y en la divulgación sobre la cosmovisión de salud mapuche a la población en general. Para la encargada del Programa PESPI, la función cumplida por la facilitadora era clave al interior de los centros de salud primaria pues orientaba a las personas que buscaban atención y en algunos meses enseñaba o mostraba algunas temáticas de salud mapuche; no obstante, este trabajo no incluía necesariamente a los profesionales de los distintos programas.

“*El rol de la facilitadora intercultural es potente en los centros de salud, ahí en la presencialidad, acogiendo, acompañando a toda persona usuaria que se acerca y que requiere atención* [...] *ella está todas las mañanas en los centros de salud otorgando este acompañamiento, acompaña y también por ahí, hay algunos meses en que lleva algunas temáticas para intervención en salas de esperas, no sé, hierbas medicinales, la cultura, la cosmovisión*”. (Encargada comunal)

Por otro lado, para la facilitadora intercultural su rol al interior del centro de salud requería de más recursos y de formalización de un trabajo continuo y remunerado. Esta falta de recursos humanos implicaba que no existía una relación entre trabajadores que permitiera avanzar en conocerse, establecer una relación profesional de confianza para así avanzar a un encuentro de cosmovisiones.

“*No puedo llegar al CESFAM y decir ‘mándenme tantos pacientes semanales’, yo soy muy cuidadosa, en este caso el médico o el doctor o matrona tiene que conocernos a nosotros primero y conocer nuestro programa de qué se trata y conocer el pueblo originario* [...] *ese es nuestro objetivo: trabajar colaborativamente o en grupo*”. (Facilitadora intercultural)

“*Hablando sinceramente, más recursos, recursos para los facilitadores, porque yo con una tarde no alcanzo a hacer todo, ni me alcanzan a conocer todos. Es la mala política podría decirlo, porque tampoco tenemos un bienestar* [...] *porque es un aporte el que nos dan a nosotros, ni siquiera un sueldo. Tampoco es de todo el año, no es tener un trabajo, trabajo estable o trabajo digno*”. (Facilitadora intercultural)

#### Coexistencia e independencia de los sistemas de salud

Tanto para las y los profesionales, como para las personas usuarias participantes, el conocimiento sobre la salud mapuche no era profundo, solo destacaban algunos elementos, como la importancia del uso de hierbas. La salud mapuche y la medicina occidental se reconocían como sistemas de salud independientes, diferentes en cuanto a los sistemas de validación de sus prácticas. Por un lado, la medicina occidental se validaba en las ciencias y por tanto se estudiaba en la educación superior y, por otro, la salud mapuche sería más bien un proceso de socialización entre generaciones y la experiencia.

“*La medicina generalmente en la cultura mapuche tiene muchas creencias en la medicina de hierbas*”. (Persona usuaria mapuche)

“*Nosotros estamos tan apegados con ese modelo biomédico y para ellos no es tan así, porque el machi no es alguien que estudie, universitario, sino que es un aprendizaje que ellos van transmitiendo a través de las generaciones y que finalmente funcionan, pero por las experiencias que visualizan*”. (Profesional de kinesiología-fisioterapia)

Ante este escaso conocimiento, un profesional proponía que el funcionamiento de ambos sistemas podría ser en paralelo, ya que las personas acudirían a uno u otro según sus preferencias. Y en el caso de fracasar con el uso de hierbas, se podía comunicar al personal médico sobre estas prácticas, con una apertura a la recepción de dicha información y el análisis sobre la interacción que pudieran tener con el uso de medicamentos, desde principios de la farmacología. En este sentido, la medicina mapuche sería conocida por los profesionales de salud, pero aún analizada desde la cosmovisión científica de la salud-enfermedad.

“*Hay gente que no va al médico, sino que se prefiere utilizar sus medicinas que es la hierba, el ungüento, cuando después de que la enfermedad o que esté padeciendo no sé cure y vayan al médico sepan transmitir esta información y la persona que esté ahí, en este caso el médico, sepa recibir esa información. Sepa que tiene que hacer con que la persona utilice tal cosa frente a un medicamento que a lo mejor puede ser sinérgico, puede amplificar el efecto*”. (Profesional de kinesiología-fisioterapia)

Adicionalmente, las personas usuarias también visualizaban un sistema de salud mapuche que no funcionaba frente a todas las enfermedades y, por tanto, deberían acudir al otro sistema, que contaba con mayor precisión científica. Esta comprensión dificultaba la pretensión de diálogo, pues existía una asimetría entre los sistemas de salud, en este caso atribuido a la mayor efectividad del sistema occidental.

“*Si dan tratamiento para la enfermedad los mapuche, los machi que le llaman, dan jarabe que se yo, desde la misma hierbas, ponen en tratamiento a las personas, pero hay enfermedad que tampoco pueden sanarlas, tiene que ser ayuda de un médico que sepa bien la enfermedades o la vacuna que se yo, es más seria la cosa* [risa...] *más exacta*”. (Persona usuaria mapuche)

## DISCUSIÓN

Las experiencias de este estudio reflejan una marcada brecha en la incorporación de la interculturalidad en las prestaciones relacionadas con la rehabilitación, en las que se incorpora la actividad física como estrategia de tratamiento. El caso estudiado muestra la implementación de un programa de salud intercultural que se ha centrado en el fortalecimiento de las organizaciones y el rescate de las prácticas de salud mapuche, no otorgando importancia al trabajo integral con otros programas del sector salud. En la práctica, los programas funcionan de manera independiente, existe escaso conocimiento por parte de los profesionales de la salud sobre el PESPI y, por tanto, los profesionales de kinesiología-fisioterapia no ven necesario instalar ajustes para trabajar con pertinencia cultural. 

Las experiencias y percepciones relacionadas con la interculturalidad en actividad física se ven condicionadas por la distinción entre dos categorías de personas mapuche, basadas en la mantención o no de costumbres y tradiciones, generando atenciones e intercambios en salud supeditadas a esta clasificación. Por lo tanto, los ajustes realizados por las y los profesionales de kinesiología-fisioterapia son reducidas a la condición de salud de la persona, dejando de lado la consideración cultural. También las personas mapuche coinciden con la distinción de categorías, y algunas de las personas entrevistadas se ubican más bien distantes de sus tradiciones culturales, por lo que tampoco demandan ajustes o mayor pertinencia cultural en las prestaciones de salud recibidas.

Por otro lado, las personas mapuche entrevistadas han sufrido discriminación y exclusión, lo que generó menor participación en instancias comunitarias de salud y ocultamiento de sus ideas y pertenencia étnica que, en términos de atención y rehabilitación física, afecta en la adherencia al tratamiento y la mantención del cuidado^17^. Pero, además, da cuenta de cómo las condiciones de salud de las poblaciones están determinadas por las asimetrías de poder y las discriminaciones presentes en la sociedad, siendo la discriminación racial y étnica una de las más relevantes y generalizada. Este tipo de discriminación tiene como antecedente las desventajas acumuladas a través de generaciones y la herencia generada por la esclavitud, el colonialismo, el imperialismo, el ultranacionalismo, el absolutismo étnico y la xenofobia^37^. Estos resultados coinciden con otro estudio que señala que las personas mapuche han vivido discriminación en la atención de salud, presentando mayor malestar físico y emocional que el resto de la población, asociado a sentimientos negativos como la vergüenza, la desesperanza y la sensación de pérdida cultural^38^. Es por ello que se ha caracterizado al origen étnico y el racismo como determinantes sociales de la salud, que interaccionan con otros determinantes (estructurales e intermedios). Están presentes en todo tipo de sociedades, gobiernos e instituciones, manifestándose en leyes, políticas, distribución de recursos e incluso en las prestaciones de salud^37^.

Por lo tanto, parece ser que el sistema de salud no cuenta con las oportunidades para promover la valoración cultural de este pueblo originario. En contraparte, en este caso estudiado, la valoración colectiva de las actividades de la comunidad mapuche, de su cultura y sus saberes, permitiría reconocer su valor y aporte a la salud y al empoderamiento de quienes participan. En este sentido, una primera etapa para la implementación del PESPI, centrada en el fortalecimiento de las organizaciones mapuche, parece ser necesaria para promover la satisfacción con la pertenencia étnica a partir de la resignificación de una identidad colectiva y la percepción positiva de esta identidad^38^. El PESPI es un avance en cuanto al rescate de la salud de pueblos originarios y la reconstrucción del sistema de salud mapuche en contextos urbanos^39^; no obstante, se requieren otros esfuerzos para cambiar el racismo estructural presente en el sistema de salud, desde la concepción de salud y del sistema de salud y la formación de profesionales en competencias interculturales^40^. 

En este estudio emerge la advertencia de que la interculturalidad puede ser malinterpretada y utilizada para la folklorización, es decir, caracterizar las tradiciones indígenas desde la visión occidental, promoviendo un uso instrumental de las costumbres mapuche sin una apropiada comprensión cultural. La folklorización ha sido debatida como un continuo de la colonización, que produce ahistorización, fragmentación y discriminación, seleccionando y desconectando algunas prácticas en favor de un proyecto determinado^41^. Por otro lado, esta selección de prácticas culturales puede ser considerada como una estrategia de expresión de la agencia indígena para recuperar prácticas culturales e interpelar a los discursos sobre extinción indígena^42^. Esto último puede ser considerado en las prácticas de salud intercultural, pues se rescatan acciones de cuidado no necesariamente conectadas a las demandas históricas de recuperación territorial del pueblo mapuche; no obstante, permite el encuentro y el rescate cultural de la salud mapuche en el contexto urbano. Asimismo, estas prácticas de salud podrían conducir a una burocratización de las acciones de salud indígena y a la comprensión del enfoque de la interculturalidad como una nueva forma de colonización^43^.

Es fundamental tener presente que los sistemas de salud cumplen un rol fundamental en la protección de los derechos humanos, la mitigación de las desigualdades en materia de salud, y pueden ser utilizados como plataformas para una acción social más amplia destinada a combatir el racismo y la discriminación racial^37^. 

## CONCLUSIÓN

La pertinencia cultural en la atención kinésica-fisioterapéutica en el caso estudiado no existe, las y los profesionales del área no poseen conocimientos sobre cómo brindar una atención que promueva el diálogo intercultural, ya que no cuentan con formación ni oportunidades de conocer y trabajar colaborativamente con el programa de salud indígena. Además, este último cuenta con escasos recursos para cumplir sus funciones, lo que produce precarización laboral de las facilitadoras interculturales y una priorización del trabajo con la población usuaria. Aspectos locales que permiten comprender los desafíos para incorporar la pertinencia cultural. Sin contar las experiencias de discriminación que viven las personas mapuche, que conducen a la asimilación cultural y la no exigencia de una salud pertinente. 

A pesar de la existencia de un enfoque de interculturalidad en la atención primaria, no existe una transversalidad en su implementación. Esto podría explicarse por el funcionamiento segmentado y no articulado de los programas de rehabilitación y salud indígena; por la falta de competencias en las y los profesionales para implementar los enfoques con que se compromete el sistema de salud; y por el poco cuestionamiento sobre las ideas de igualdad o trato igualitario que operan en la atención de salud, pues no necesariamente una práctica homogénea implica igualdad en el derecho a la salud y puede invisibilizar discriminaciones estructurales en las prácticas de salud. 

Las experiencias relatadas sobre ocultamiento de las personas mapuche no son una característica de su personalidad identificada como timidez, más bien reflejan una forma de resistencia para afrontar las asimetrías de poder entre culturas, que ha llevado a la asimilación cultural, particularmente en el contexto urbano. En consecuencia, ni los profesionales, ni las personas usuarias consideran como una necesidad o demanda implementar ajustes u otras formas de diálogo intercultural en la práctica kinésica-fisioterapéutica.

La incorporación de ajustes con consideración cultural dentro de la atención de salud debe ser respetuosa, pertinente y contextualizada en las creencias de cada pueblo, para evitar la apropiación de prácticas, su implementación sin sentido o su omisión. Es importante avanzar en el diálogo intercultural en salud, pues el reconocimiento y valoración de la cosmovisión indígena influye en el fortalecimiento de la identidad cultural y el buen vivir. Se deben explorar distintos niveles de vinculación entre programas de salud, como los de rehabilitación y pueblos indígenas, y se requiere una continua reflexión y diálogo para identificar los modos de articulación respetuosa y que pongan en valor las diferentes culturas en una atención centrada en las personas y sus comunidades.

Este estudio es una contribución novedosa en el ámbito de las perspectivas culturales de la salud y la rehabilitación, que busca incorporar el concepto de equidad en el acceso a la salud, y el tipo de prestaciones otorgadas a poblaciones culturalmente diversas y que han sido vulneradas en sus derechos al acceso a la salud. En cuanto a las limitaciones este estudio, no tiene pretensiones de representatividad, dado que en cada comuna las condiciones y estrategias de implementación del PESPI son distintas, por lo que sugerimos una lectura situada de estos hallazgos. Tampoco se incluyó análisis documental que permitiera contrastar la implementación de la política y su contenido. Se requieren nuevos estudios para comprender y diseñar modos de atención en salud que incorporen la interculturalidad en contextos rurales y urbanos, que aporten a disminuir las brechas de salud entre los distintos pueblos. 
