# Supplementary material for: Desafíos para la pertinencia cultural de fisioterapia en la atención de población mapuche en Chile
Source: Salud Colect. 2024 Oct 22;20:e4849. [Article in Spanish] doi: 10.18294/sc.2024.4849 (PMC11925734; doi:10.18294/sc.2024.4849)
Supplement: Translation

# Translation: Challenges for cultural relevance of physical therapy in the care of the Mapuche population in Chile

**Authors:** Mónica Manríquez-Hizaut, Trinidad Martínez-Campos, Nicolás Lagos-Gallardo, Jame Rebolledo-Sanhueza

**Affiliations:** 1 Master in Public Health. Assistant Professor, Department of Physiotherapy, Faculty of Medicine, Universidad de Chile, Santiago, Chile. mmanriquezh@uchile.cl; 2 Degree in Physiotherapy. School of Physiotherapy, Faculty of Medicine, Universidad de Chile, Santiago, Chile. trinidadmartinez@ug.uchile.cl; 3 Degree in Physiotherapy. School of Physiotherapy, Faculty of Medicine, Universidad de Chile, Santiago, Chile. nicolaslagos@ug.uchile.cl; 4 Corresponding author. Master in Community Psychology. Assistant Professor, Department of Physiotherapy, Faculty of Medicine, Universidad de Chile, Santiago, Chile. jame.rebolledo@uchile.cl

**Keywords:** Cultural Competency, Physical Therapy, Indigenous Peoples, Exercise, Chile

## Abstract

Physical therapy rehabilitation must address the cultural preferences of the population, and as its main tool, physical activity should be analyzed from an intercultural health approach. This study aims to characterize the experiences of physical therapy care related to physical activity from an intercultural perspective. Using a qualitative approach, between June 2021 and March 2023, eight semi-structured individual interviews were conducted with adult Mapuche individuals, professionals in the field, and workers from the Indigenous Health Program in primary care centers in an urban municipality in Santiago, Chile. There are challenges to implementing interculturality, such as the gap between health programs, lack of professional training, and discrimination against the Mapuche community. In rehabilitation services, particularly in physical activity practices as a treatment strategy for Mapuche individuals, cultural relevance is limited or nonexistent.

## INTRODUCTION

The concept of health has a broad definition that spans from a biomedical approach to a cultural dimension. The World Health Organization (WHO) defines health as “a state of complete physical, mental, and social well-being, and not merely the absence of disease or weakness”.^1^ Although the state of complete well-being may be considered unrealistic, this understanding has allowed health and illness to be viewed as a continuum and a state of ongoing balance, encompassing various biological and social factors in constant interaction and production.^2^ From a cultural perspective, it is understood that all communities develop different health models to address illnesses and maintain or restore health.^3^

Collective health addresses the health-illness continuum from a historical and situated perspective, accounting for how social groups organize health services, including the integration of various forms of knowledge,^4^ among them the health views of different Indigenous cultures. Menéndez^5^^)^ argues that through the processes of health and illness, most ideological-cultural conflicts are revealed, and social conflicts are made more transparent and less biased, often analyzed from other perspectives. In other words, by observing the dynamics present in health-illness phenomena, we are also investigating the power asymmetries present in society, such as ethnic or gender inequalities. Therefore, it is essential to understand how interculturality in health is developed, which is considered a framework of action aimed at promoting equal treatment among different cultural groups, with health regarded as a priority and a fundamental right. Furthermore, it involves the capacity of health professionals to integrate scientific knowledge with traditional beliefs and practices when addressing a health-illness situation.^6^

The Pan American Health Organization has established interculturality in health as a priority for Indigenous peoples in Latin America, promoting the development of health policies guided by principles of comprehensive care, sovereignty of peoples, cultural revitalization, and reciprocity in relationships.^7^ In Chile, interculturality in health is recognized as a cross-cutting principle within the health system, especially in primary health care (PHC), which is managed locally through territorial and family and community health approaches.^8^ In this context, greater openness to intercultural dialogue would be expected; however, despite the positive regard PHC professionals may hold for Indigenous health, they recognize asymmetries between health systems and uncertainties regarding their complementarity.^9^

Currently, the Chilean State recognizes ten Indigenous peoples: Mapuche, Aymara, Rapa Nui, Atacameño, Quechua, Colla, Diaguita, Kawashkar or Alacalufe, Yámana or Yagán, and Selk’nam, representing 12.8% of the national population, which is slightly more than two million people. The Mapuche population is the largest, accounting for 79.84% of the Indigenous population and residing mainly in the Araucanía and Metropolitan regions.^10^ The Indigenous population faces a high degree of inequality, reflected in higher general mortality rates compared to the non-Indigenous population.^11^ Economically, the Indigenous population experiences a higher poverty rate (8.8%) and extreme income poverty (2.7%) compared to the non-Indigenous population (6.2% and 1.9%, respectively).^12^ There are also inequalities in employment quality and education; for example, the average years of schooling for the Indigenous population is 10.3 years, while the non-Indigenous population averages 11.3 years. Additionally, Indigenous individuals show a higher affiliation with the public health system (86.4%) compared to the non-Indigenous population (77.2%).^13^

Mapuche health embodies a worldview of life and well-being that considers ancestral origins, spirituality, and balance with nature; thus, the disruption of this balance is manifested as illness.^14^ This Mapuche health-illness model is practice by traditional healers who do not follow a standardized training as in the biomedical model but instead acquire a gift through dreams, visions, and a deep connection with nature and ancestors.^15^

Given this cultural specificity, Indigenous people sometimes distrust Western medicine, which may exclude them from health promotion programs. Conversely, disconnecting from traditional practices has been shown to lead to poorer physical, mental, and overall well-being.^16^ Additionally, the stigmatization and discrimination against Mapuche individuals are exacerbated within healthcare settings, where it is reported that 82% of Mapuche adults feel disdained and treated as inferior within health services.^17^

With the aim of reducing the gap between Indigenous and Western health systems based on mutual respect and equal recognition of their knowledge systems, intercultural health is proposed as a set of policies and actions to understand and incorporate the culture of individuals in the care process,^18^ through the recognition of health beliefs to advance knowledge dialogue.^19^ The intercultural approach seeks to achieve health equity, understood by international organizations as the “pursuit of fairness and justice by eliminating differences that are unnecessary and avoidable”.^20^ However, comparative studies in Latin America^21^ indicate that attempts to incorporate cultural approaches within the health system have not been effective, revealing institutionalized racism across various sectors of health care systems.

In Chile, the Ministry of Health recognizes intercultural health as actions developed between health teams and intercultural facilitators to “meet accessibility needs, timeliness in morbidity care, adaptation of health organization, and culturally appropriate health care for the needs of Indigenous peoples”.^22^ The first intercultural health program was established in 1992, with its core focuses on training, research, community knowledge, and teaching. In 2008, the Special Program for Health and Indigenous Peoples (PESPI) was created to consolidate an intercultural health system that acknowledged the limitations of medical systems in addressing current health issues, recognizing the existence and validity of other healing systems, such as Indigenous ones. This program has three main pillars: equity, an intercultural approach, and Indigenous social participation.^23^ Various studies have evaluated this policy, characterizing its implementation as a complex, heterogeneous space with multiple tensions.^24^^,^^25^^,^^26^^,^^27^ However, there are no specific national studies on interculturality in the field of rehabilitation or physiotherapy.

Sports and physical activity are valued practices in the field of physiotherapy and are recognized for their health benefits.^28^^,^^29^^,^^30^ For Indigenous cultures in Central and South America, these physical activities convey the myths and cultural values that connect the material and immaterial worlds of each community^31^ Physical activity and sport can be seen as polysemic and cultural concepts, historically situated, and therefore relevant when considering rehabilitation from an intercultural approach. A study with Māori women demonstrated that taking ethnic preferences into account, such as modifying the environment for various activities, increased their participation and adherence to physical activity guidelines.^32^

For physiotherapy, a discipline that focuses on human movement and its relationship with the external environment, sport and physical activity are significant and prominent in health care. Moreover, the rehabilitation programs developed in the primary care sector of the Chilean public health system should operate from an intercultural approach, which has not been explored to date. Expanding knowledge regarding the cultural relevance of practices in physiotherapy services will contribute to improving the comprehensiveness of care and its cultural appropriateness. The aim of this study was to characterize the physiotherapy care experiences related to physical activity of Mapuche adults, professionals in the field, and workers from the Indigenous health program at primary care centers in an urban community in Santiago, Chile, from an intercultural perspective.

## METHODOLOGY

A qualitative study^33^ was conducted on the cultural relevance of physiotherapy care for Mapuche individuals in an urban area of Santiago, Chile. It should be clarified that in this research, the term “Mapuche” is used for both singular and plural, as the word in Mapudungun contains the plural “mapu” (land) and “che” (people).

The participants included in this study were working in primary health care (PHC) programs, Mapuche individuals using PHC services, and key agents from the PESPI program who participated in its community implementation. We established a profile for each key agent based on the following inclusion criteria: physiotherapist in PHC services with at least one year of experience, working in the musculoskeletal and/or respiratory areas. The group of key agents from the PESPI program had to be individuals in leadership, management, and/or guiding roles in areas of interculturality and cultural relevance within the PHC service. Finally, the users had to identify as belonging to the Mapuche people and have received physiotherapy care in PHC in the last two years. Participants for the study were recruited based on each profile. 

The final number of participants was eight individuals (Table 1), defined through theoretical sampling according to the information produced in the interviews, aiming to identify themes and experiences to explore further, which guided the recruitment of participants. This final composition met saturation criteria, understood as the point at which the research team has sufficient elements to construct a comprehensive, novel, problematizing, and convincing theory on the topic^34^.

**Table 1:** Sociodemographic characterization of the participants. Santiago, June 2021 and March 2023.

Sociodemographic variables	Mapuche users	Physiotherapist	Key agents of the Special Health and Indigenous Peoples Program (PESPI)
**Sex**			
Male	1	1	0
Female	2	2	2
**Age (average in years)**	61	34	45
**Indigenous ancestry**			
Yes	3	0	1
No	0	3	1
**Years of work experience in the community (average)**	Not applicable	7	10

Source: Own elaboration.

The recruitment was carried out using a gatekeeper strategy, where individuals who had contacts matching the desired participant profile mediated the invitation. Subsequently, the research team formalized the invitation and conducted the informed consent process. Of the individuals invited to participate, only one declined due to lack of availability.

Data collection was conducted through individual semi-structured qualitative interviews, led by a duo from the research team, consisting of a third-year physiotherapy student and a tutor (academic) from the Department of Physiotherapy. Prior to the start of the fieldwork, a pilot interview was conducted as a training exercise for part of the research team in training and to test the interview guide.

The interviews were guided by a set of open-ended and flexible questions, which directed the information production process expressed in the verbal and non-verbal responses of the interviewee^35^. The interview guide began with a series of identification questions regarding the individuals, their relationship with the Mapuche people, and their beliefs about the health and illness process. Then, it explored practices for addressing or adjusting cultural preferences in care and their considerations within physical activity. Finally, participants were asked about their perception of the implementation of interculturality in PHC, along with a space for additional comments, questions, and concerns. The guide was analyzed after each interview, incorporating changes to assist the training researcher. The total number of interviews was conducted between June 2021 and March 2023 to finalize the theoretical sampling. All interviews were conducted via video calls using an institutional Zoom account and lasted between sixty and ninety minutes. These sessions were recorded in audio and then transcribed verbatim.

Qualitative content analysis was conducted, as it allows for an in-depth interpretation of the collected data and explains complex social phenomena.^36^ This methodological orientation provides systematic procedures for describing the content of the data, with the aim of inferring knowledge related to their production conditions. Each interview was analyzed by two independent researchers using a coding strategy.^36^ A deductive analysis was performed, guided by the main themes of the interviews, followed by an inductive analysis considering the emerging themes. The procedure involved assigning a common designation to a set of data sharing the same idea, in order to discover relationships and begin coding, generating condensed units of meaning that were labeled to form codes. These codes were then grouped into categories based on their similarities. Finally, the properties included within a category were labeled and divided into dimensions for analysis.^36^ The team collectively defined the dimensions that represented the main results of the study. Data management and the coding process were supported by the qualitative analysis software ATLAS.ti.

This study was approved by the Ethics Committee for Research on Human Beings of the Faculty of Medicine at the University of Chile (Act No. 008/10, May 2022), and all participants provided their consent. Additionally, the research team conducted a results feedback session with staff from the PESPI program and local authorities to present the findings of this research, aiming to contribute to health care perspectives from an intercultural approach.

## RESULTS

The findings of this study are organized into dimensions that explain the lack of implementation of interculturality in rehabilitation services within the context of primary care in an urban community. We begin with a presentation of the case and the relevant relationships for the phenomenon under study, considering the organizational context of the local health system. Subsequently, we address perceptions of interculturality in physical activity practice and specifically in the health system and physiotherapy care.

### Characterization of the case study

The case studied is situated in an urban community of the Metropolitan Region, specifically involving activities implemented among municipal programs of a community health department. The physiotherapy professionals work in the clinical field, particularly in rehabilitation and physical activity. Regarding the key agents of the *Programa Especial de Salud y Pueblos Indígenas* (PESPI) [Special Health and Indigenous Peoples Program], one of them coordinates the implementation of the Program, while the other acts as an intercultural facilitator and is also the president of a Mapuche organization in the community. Finally, the interviewed users identified themselves as Mapuche descendants who received physiotherapy care at one of the Primary Care Centers in the community between 2021 and 2022. One of these individuals was also linked to PESPI.

Rehabilitation and health promotion programs do not have a specific focus on working with indigenous populations; however, they are developed within the framework of primary health care using a comprehensive and community-based care model, which includes interculturality as an action approach. The professionals in these programs, in addition to not being direct descendants of the Mapuche people, report lacking personal experiences with their cultural practices. 

The PESPI program develops activities focused on Mapuche health, strengthening indigenous organizations in the commune, and providing training to the general public and health professionals. The program coordinator also lacks close personal experiences; however, due to her role, she has become familiar with Mapuche health practices and the work of indigenous organizations in the area.

On her part, the intercultural facilitator works sporadically in the PESPI program and was born and raised in her Mapuche family according to the traditions of her people. Her first language was Mapudungun, which she still uses today. In addition to her knowledge of the language, she attributes a significant part of her work to her life experience in the environment and practices of her people:

*“I was born and raised in the countryside, and fortunately, I was born by the fireside. I have all the care typical of our Mapuche culture and the knowledge of my first native language, Mapudungun, which I currently speak very well. Having all those connections with the land, with nature, the animals, the birds, the river, the hill, and the crops - all those wonders - is why today I share that knowledge.”* (Intercultural facilitator)

In the case of the users, despite some having been born in rural areas within families with Mapuche customs, these traditions were fading as older generations passed away and younger generations migrated to the cities. They attributed this to a significant disconnection from cultural practices, which they viewed negatively:

*“I feel a sense of belonging to the Mapuche people from the way I got lost; I think that happens to a lot of people because there is so much disconnection. I am from Warria, or rather, we call the city Warria* [...] *there are things that slip out of our hands, bad decisions. Many times, we feel disconnected, disconnected from the land.”* (Mapuche user)

Two users reported that, in their life in the city, they felt the need to reconnect with the practices of their people, either by returning to the communities where they were born or by approaching indigenous organizations that aimed to revive those practices. In the case of this study, these organizations were part of the PESPI program, which had developed Mapuche health practices from within the organizations themselves.

The work of Mapuche organizations in the urban area was considered key for individuals to reconnect with the practices of their people and to develop them in connection with their current environment. For the intercultural facilitator, this encounter with the Mapuche organization in the urban territory was crucial for valuing her own wisdom and recognizing cultural knowledge and practices:

*“...I was strengthening my kimün, our wisdom, and being able to meet with a group of my own brothers from my people who started to value... you know a lot, you are very special, you know how to make sopaipillas, prepare mate, so that began to lift me up* [encourage me]*.”* (Intercultural facilitator)

### Perception of interculturality in physical activity

The perception of the need to include interculturality in physical activity practices is mediated by the understanding of what it means to be Mapuche in the city, experiences of concealment and ethnic discrimination, and the lack of training and questioning by the professionals involved in the case. Additionally, potential misinterpretations of interculturality emerge within the current context of physical activity practice in physiotherapeutic care. The following results address these findings.

#### Being champurria: understanding “being urban Mapuche”

People in their narratives described the existence of two types of Mapuche individuals. The first is based on the persistence of customs and traditions and being originally from the southern region, which would be reflected in the use of the Mapudungun language or in being a descendant of two Mapuche parents, characterized as “real” Mapuche. The second is what they referred to as “champurria,” either as a result of mixed heritage or by being born in the city, without growing up in contact with Mapuche cultural practices. This distinction was identified by rehabilitation professionals to justify different forms of interaction:

*“There are people who are of Mapuche origin, but they are more accustomed to current society, unlike other people who follow their rituals and culture, so the relationship is with two different people.”* (Physiotherapist)

*“We have not had Mapuche users, Mapuche, Mapuche, who speak only Mapudungun... but rather they are people who are Mapuche, who know Spanish and communicate in Spanish.”* (Physiotherapist)

The quotations show the idea of two categories of Mapuche individuals with different approaches to their culture. This difference is also recognized by the intercultural facilitator and the service users.

*“There are Mapuche people here, but urban, who didn’t know many things but had all the willingness.”* (Intercultural facilitator)

*“I am champurria, born and raised in Santiago; I have a different concept of life, let’s say, I am more winka than Mapuche.”* (Mapuche user)

This distinction will have implications in health practices, as for professionals, the need for adjustments is not evident, and for service users, who are familiar with the Western and urban health system, there will be no need for cultural adaptations in physical activity care.

#### Concealment due to experiences of discrimination

Among the three participant groups in the case, a common practice of concealment was identified, which some understood as shyness on the part of the Mapuche population. However, based on the intercultural facilitator’s life history, these practices could be interpreted as concealment strategies to avoid discrimination or as a result of discrimination.

*“They are rather more timid, more reserved people, who are not always going to say it out loud, or even to oneself... you find out quite indirectly, so to speak, that the person is Mapuche because it’s not something they are going to verbalize in a workshop, for example* [...] *they try to make it go unnoticed so that there are no differences with them, so that they won’t be attacked, because they are always on the defensive.”* (Physiotherapist)

The Chilean school system was identified as one of the institutions where rejection of Mapuche culture and discriminatory practices had been generated, due to the hegemonic imposition of the prevailing Western culture in Chile. Specifically, this included prohibiting the use of the Mapudungun language and ethnic discrimination through ridicule related to physical appearance-an experience that both the intercultural facilitator and her daughters went through, leading them to feel that being Mapuche was a problem they needed to distance themselves from.

*“I faced a great deal of discrimination for being Mapuche or for being a woman, everything, because I had the first language that gave me my Mapuche soul; it was also hard for me, as it is for anyone to learn another language and be able to speak it well... school treated me very poorly, first because it took away the original language that I had, which is my life, my way of living, so that marked me a lot in my life and for a long time I suffered from that discrimination, from pains in my heart, discrimination for being Mapuche, because their hair is like that, because their face is like that* [...] *I didn’t want to know anything, not even about my last name because I was very hurt.”* (Intercultural facilitator)

Service users also mentioned experiences of discrimination or feeling ashamed, and thus acknowledged concealing or feeling aversion toward their Mapuche surnames as symbols of identification, even though, through their own experiences of reconnecting with their ethnic roots, they felt this was something gradually being left in the past.

*“The Mapuche always felt ashamed of the Chilean, of their way of speaking, their way of walking, I don’t know, so they were very shy, but now, that has changed.”* (Mapuche user)

Issues like this led to Mapuche people being rendered invisible and marginalized from collective practices carried out in primary healthcare (PHC), even causing them to avoid participating in such activities. Even when involved, they did not feel they could share their preferences, making it difficult to implement intercultural spaces that would allow both Mapuche and non-Mapuche people to recognize and value the Indigenous worldview, thereby overcoming concealment practices. This collective appreciation was key for the intercultural facilitator, enabling her empowerment and reconnection with her own culture, as well as its forms of expression and opinion, which allowed for full and integral participation across various areas of primary healthcare to achieve the implementation of intercultural health.

*“Today I stand strong, I am flourishing, I am bearing the fruits of my life as a Mapuche, not ashamed of my clothing, and I hope everyone can look at me and turn around to see me because of my clothing that catches their attention, and not like in the past when I used to dress.”* (Intercultural facilitator)

#### Lack of critical reflection and professional training

When speaking with rehabilitation professionals about the need to adjust care practices and physical activity from an intercultural perspective, it became a challenging topic to address due to their lack of experience in this area. Although some were able to identify differences among service users in terms of performing specific physical exercises and recreational activities, as well as working in groups, they did not see a need to inquire about users’ preferences. On the other hand, they noted that the only adjustments made related to individuals’ biological conditions, as they adhered to professional criteria within Western health practices.

*“It’s hard to get them involved, it’s hard to encourage them to participate; they are not used to exercising recreationally, but they are more adapted to functional exercise like feeding animals. It’s difficult to engage them in that entertainment aspect, but they are indeed active.”* (Physiotherapist)

*“It adapts depending on the person’s physical condition, but there is no differentiation* [...] *I wouldn’t dare to say there is a separation by indigenous people, but rather based on the situation the person is in. For example, if a person has osteoarthritis, there are certain exercises they cannot do; in another context, it’s just adaptation depending on the biological condition the person is in.”* (Physiotherapist)

On the other hand, even when professionals could envision ways to incorporate healing practices into therapy, they acknowledged that there were no mechanisms in place to facilitate such requests.

*“I haven’t had anyone ask me to end their therapies in a different way. I find it difficult; if they wanted to communicate it to me, they are almost always in group activities because we try to move on to workshops quickly.”* (Physiotherapist)

Finally, during the conversation, professionals considered it possible to introduce adjustments that would allow for relevant care. Some questioned the biomedical or clinical relevance of such interventions more critically, while others focused on understanding the processes of disease awareness and utilizing resources from the Mapuche worldview. For instance, the “self-knowledge” that individuals developed to be attuned to their bodies and prevent illnesses was valued. On the other hand, there was an acknowledgment of a lack of knowledge on how to implement interculturality, and they perceived that differentiated activities were not conducted to avoid discrimination. One participant suggested that the same level of care implied the same quality; therefore, any adjustments would only be necessary in the clinical realm based on physical condition. Another participant perceived both positive distinctions and negative distinctions, indicating that, under the notion of equality as homogeneity, culturally distinct customs could be transgressed or at least not considered.

*“Attention is only adapted depending on the physical condition of the person, but there is no differentiation... I believe that, in general, all people should be treated the same, with the same passion and the same quality.”* (Physiotherapist)

*“We could make distinctions, but in a positive way, never a distinction from a negative perspective. Perhaps if you want a different type of care, and since we are not aware, maybe we need that space. And in this stance of treating everyone as equal, and that everyone must participate equally, we are overlooking and transgressing something that is part of their rituals and customs. So, in that sense, it would be very enriching to learn and understand in order to improve.”* (Physiotherapist)

#### Misinterpretation and folklorization from interculturality

Participants with a stronger connection to the Mapuche people - those who were knowledgeable about their worldview - recognized differences in the practices and uses of physical exercise. They did not see these practices as related to health or treatment methods; rather, they viewed them as everyday physical activities associated with ceremonial occasions linked to community life.

*“So the Mapuche people don’t have that concept of mobility, moving the arm, moving the legs, doing this; we don’t have that culture.”* (Mapuche user)

*“*[It’s not] *about doing exercise because I want to feel good, or because my body needs to exercise. The Mapuche people don’t see it that way. All the exercises that happen are through ceremony* [...] *a game of chueca or palin, as we call it; sharing a moment of play is a ceremony for us. So we accompany it with music; the trutruca, you are constantly playing the kultrún; it’s a way of exercising, but in a Mapuche context. We don’t do it just to say, for example, because I want to lose weight.”* (Intercultural facilitator)

The practice of play or physical exercise was embedded in a context of cultural practice, community care, and spiritual development, rather than individual care. This did not align with the understanding of exercise prevalent in Western culture and among the physiotherapist themselves. One user, seeking to bridge both practices, proposed using traditional Mapuche game implements as training elements for the “kinesiological movements” in rehabilitation treatment.

*“They take the palín, do you know this? The palín. So take the palín, move it, hip movements, move the palín over there, move the palín over there, do you see it or not? They can do it; there are many movements that are physiotherapy-related, which they can use as tools of the Mapuche people.”* (Mapuche user)

The intercultural facilitator was emphatic in stating that cultural relevance was not achieved simply by instrumentalizing exercise with implements specific to Mapuche traditions, as these did not necessarily follow the logics and purposes of Western culture. Instead, they served as a means for the transmission of cultural and ceremonial rites. Therefore, incorporating these tools without a cultural sense would violate the principles of respect and appreciation proposed by the intercultural approach.

*“Our dance or another game is done barefoot* [...] *there’s a reason for this because our connection with the earth is fundamental; the foot must have that contact with the ground. That’s why we often perform dances on the earth, not on cement. If I’m asked to hold a ceremony on cement or in an apartment or in a place that isn’t suitable, I won’t do it because, in that case, I would be folklorizing or selling my culture.”* (Intercultural facilitator)

### Perception of the health system and physiotherapeutic care

Other factors explaining the lack of interculturality in physiotherapy care were related to the lack of communication and collaboration between the Indigenous health program (PESPI) and other health programs. Additionally, from a place of ignorance, the Mapuche and Chilean Western health systems were viewed as parallel systems, highlighting a difference that spoke more of the coexistence of various cultural practices rather than an intercultural dialogue.

#### Distance between the special health program for indigenous peoples and other health programs

There is limited knowledge about the PESPI program among physiotherapy professionals. They acknowledged a lack of information and dissemination, as well as a lack of prioritization from leadership positions. Despite working in primary care in the same territory, they did not know beyond the names of the individuals in charge.

*“I don’t know the PESPI program* [...] *it shows that there is a lack of information and outreach because I’ve been working for three years and I’ve only heard about the program two or three times.”* (Physiotherapist)

However, professionals recognized the need for tools that would allow them to communicate effectively and connect with the culture of other individuals, including other Indigenous peoples and foreign individuals.

*“As health professionals, we should be prepared for any situation; in fact, this includes being able to communicate effectively with a person of Haitian origin, as well as with a Mapuche, Diaguita, or Aymara person, ensuring smooth communication.”* (Physiotherapist)

The PESPI program acknowledges that there are difficulties for staff to participate in training and workshops on intercultural health due to a lack of time to dedicate to these activities beyond their own work responsibilities or working hours.

*“…I believe this needs to come more from the top. We put in all the effort, but I keep saying it’s just a small stroke, very little, because yes, my desire is to provide training to all the professionals at the CESFAM* [Family Health Center]*, but that’s not the case; they would like to, but they are focused on fulfilling their work hours* [...] *maybe five will attend, but not the 30 or 50 who should be there. It’s not possible.”* (Intercultural facilitator)

On the other hand, for the community coordinator of PESPI, it was not only a matter of availability but also of willingness and political direction from the community project and health leadership as a whole. For intercultural dialogue to exist among health professionals, approval and interest from management or health direction were necessary.

“*If the person, in this case, the health director or the mayors of the municipality, is not interested in this, no matter how much I give them 24/7, it won’t work.*” (Community Coordinator)

The work of PESPI in this study focused on strengthening support for Mapuche individuals and disseminating information about the Mapuche worldview of health to the general population. For the PESPI program coordinator, the role played by the facilitator was key within primary health centers, as she guided individuals seeking care and taught or presented topics related to Mapuche health over several months; however, this work did not necessarily include professionals from the various programs.

*“The role of the intercultural facilitator is powerful in health centers, there in person, welcoming and accompanying every user who approaches and requires care* [...] *she is there every morning in the health centers providing this support, and also, during some months, she brings up certain topics for intervention in waiting rooms, such as medicinal herbs, culture, and worldview.”* (Community Coordinator)

On the other hand, for the intercultural facilitator, her role within the health center required more resources and the formalization of continuous, paid work. This lack of human resources meant that there was no relationship among workers that would allow for progress in getting to know each other, establishing a professional trust relationship, and thereby moving toward a meeting of worldviews.

*“I can’t go to the CESFAM and say ‘send me so many patients a week.’ I am very careful; in this case, the doctor or midwife has to get to know us first and understand what our program is about and get to know the indigenous people* [...] *that is our goal: to work collaboratively or as a group.”* (Intercultural Facilitator)

*“To be honest, we need more resources, resources for the facilitators, because with just one afternoon, I can’t do everything, and I can’t get to know everyone. It’s poor policy, I could say, because we don’t have well-being* [...] *because what they give us is a contribution, not even a salary. It’s not year-round; it’s not a job, stable work, or dignified work.”* (Intercultural Facilitator)

#### Coexistence and independence of health systems

For both professionals and participating service users, knowledge about Mapuche health was not deep; they only highlighted a few elements, such as the importance of using herbs. Mapuche health and Western medicine were recognized as independent health systems, differing in terms of the validation processes of their practices. On one hand, Western medicine is validated through sciences and is therefore studied in higher education; on the other hand, Mapuche health is more of a socialization process passed down through generations and experience.

*“Medicine in the Mapuche culture generally has many beliefs in herbal medicine.”* (Mapuche user)

*“We are so attached to that biomedical model, and for them, it’s not quite the same, because the machi is not someone who studies at a university; rather, it is a learning process that they pass down through generations, and it ultimately works based on the experiences they observe.”* (Physiotherapist)

In light of this limited knowledge, one professional proposed that both systems could operate in parallel, as individuals would turn to one or the other based on their preferences. In cases where herbal treatments failed, users could communicate these practices to medical staff, fostering an openness to receive such information and analyze the interaction that might occur with the use of medications from the principles of pharmacology. In this sense, Mapuche medicine would be known to health professionals, but still analyzed through the scientific worldview of health and illness.

“*Some people don’t go to the doctor; instead, they prefer to use their medicines, which are herbs and ointments. When, after the illness or suffering, they don’t get better and finally go to the doctor, they should know how to communicate this information, and the person there, in this case, the doctor, should know how to receive that information. They should understand that they need to consider what the person is using in relation to a medication that could potentially be synergistic, amplifying the effect.*” (Physiotherapist)

Additionally, service users also perceived a Mapuche health system that did not work for all illnesses, and therefore they felt they should turn to the other system, which was seen as having greater scientific precision. This understanding complicated the desire for dialogue, as there was an asymmetry between the health systems, attributed in this case to the greater effectiveness of the Western system.

*“If the Mapuche provide treatment for illnesses, the machi, as they are called, give syrup made from the same herbs and treat people, but there are some illnesses they cannot heal. It has to be with the help of a doctor who knows the diseases well or the vaccine, you know? It’s more serious* [laughs...] *more precise.”* (Mapuche user)

## DISCUSSION

The experiences from this study reflect a significant gap in the incorporation of interculturality in rehabilitation services, where physical activity is included as a treatment strategy. The case studied shows the implementation of an intercultural health program that has focused on strengthening organizations and rescuing Mapuche health practices, without placing importance on integrated work with other health sector programs. In practice, the programs operate independently, and there is limited knowledge among health professionals about PESPI; therefore, physiotherapist do not see the necessity of making adjustments to work with cultural relevance.

Experiences and perceptions related to interculturality in physical activity are conditioned by the distinction between two categories of Mapuche individuals, based on the maintenance or absence of customs and traditions. This distinction generates health care and exchanges that are subordinate to this classification. Consequently, the adjustments made by physiotherapist are limited to the individual’s health condition, neglecting cultural considerations. Furthermore, Mapuche individuals themselves align with this categorization, and some of the interviewees identify as somewhat distanced from their cultural traditions, leading them not to demand adjustments or greater cultural relevance in the health services they receive.

On the other hand, the Mapuche individuals interviewed have experienced discrimination and exclusion, leading to lower participation in community health initiatives and a concealment of their ideas and ethnic identity, which affects adherence to treatment and maintenance of care in terms of physical rehabilitation.^17^ Furthermore, this highlights how the health conditions of populations are determined by power asymmetries and the discriminations present in society, with racial and ethnic discrimination being among the most relevant and widespread. This type of discrimination has its antecedents in the accumulated disadvantages over generations and the legacy generated by slavery, colonialism, imperialism, ultranationalism, ethnic absolutism, and xenophobia.^37^ These results align with another study indicating that Mapuche individuals have faced discrimination in health care, experiencing greater physical and emotional distress than the rest of the population, associated with negative feelings such as shame, hopelessness, and a sense of cultural loss.^38^ Thus, ethnic origin and racism have been characterized as social determinants of health, interacting with other determinants (structural and intermediate). They are present in all types of societies, governments, and institutions, manifesting in laws, policies, resource distribution, and even health services.^37^

Therefore, it seems that the health system does not provide opportunities to promote the cultural appreciation of this Indigenous people. In contrast, in the case studied, the collective appreciation of Mapuche community activities, their culture, and their knowledge would allow for recognition of their value and contribution to health and the empowerment of participants. In this sense, an initial phase for the implementation of PESPI, focused on strengthening Mapuche organizations, appears necessary to promote satisfaction with ethnic belonging through the re-signification of a collective identity and a positive perception of that identity.^38^^)^ PESPI represents a step forward in rescuing the health of Indigenous peoples and reconstructing the Mapuche health system in urban contexts;^39^ however, additional efforts are required to change the structural racism present in the health system, starting from the conception of health and the health system itself, as well as training professionals in intercultural competencies.^40^

This study highlights the warning that interculturality can be misunderstood and used for folklorization, meaning the characterization of Indigenous traditions through a Western lens, promoting an instrumental use of Mapuche customs without appropriate cultural understanding. Folklorization has been debated as a continuation of colonization, producing ahistoricity, fragmentation, and discrimination by selecting and disconnecting certain practices in favor of a specific project.^41^ On the other hand, this selective adoption of cultural practices can also be viewed as a strategy of Indigenous agency, aimed at reclaiming cultural practices and challenging discourses of Indigenous extinction.^42^ This perspective can be applied to intercultural health practices, as certain care practices are preserved, though they may not necessarily be connected to the Mapuche people’s historical demands for land recovery. Nonetheless, it enables encounters and the cultural preservation of Mapuche health practices in an urban context. Likewise, these health practices could lead to the bureaucratization of Indigenous health actions and a re-interpretation of the intercultural approach as a new form of colonization.^43^

It is essential to keep in mind that health systems play a crucial role in the protection of human rights, the mitigation of health inequalities, and can be used as platforms for broader social action aimed at combating racism and racial discrimination.^37^

## CONCLUSION

Cultural relevance in kinesthetic-physiotherapeutic care in the case studied does not exist; the professionals in the field lack knowledge on how to provide care that promotes intercultural dialogue, as they do not have training or opportunities to learn and work collaboratively with the Indigenous health program. Furthermore, this program has limited resources to fulfill its functions, resulting in job precariousness for intercultural facilitators and a prioritization of work with the user population. Local aspects help to understand the challenges of incorporating cultural relevance, alongside the experiences of discrimination faced by the Mapuche people, which lead to cultural assimilation and a lack of demand for relevant health care.

Despite the existence of an intercultural approach in primary care, there is no cross-cutting implementation of it. This could be explained by the segmented and uncoordinated functioning of rehabilitation and Indigenous health programs; by the lack of competencies among professionals to implement the approaches that the health system commits to; and by the limited questioning of the ideas of equality or equal treatment that operate in health care, as a homogeneous practice does not necessarily imply equality in the right to health and may obscure structural discriminations in health practices.

The experiences shared about the concealment of Mapuche people are not a characteristic of their personality identified as shyness; rather, they reflect a form of resistance to confront the power asymmetries between cultures, which has led to cultural assimilation, particularly in the urban context. Consequently, neither the professionals nor the users consider it a necessity or demand to implement adjustments or other forms of intercultural dialogue in kinesthetic-physiotherapeutic practice.

The incorporation of culturally considerate adjustments within health care must be respectful, relevant, and contextualized to the beliefs of each people, to avoid the appropriation of practices, their implementation without meaning, or their omission. It is important to advance in intercultural dialogue in health, as the recognition and appreciation of Indigenous worldviews influence the strengthening of cultural identity and the promotion of well-being. Different levels of collaboration between health programs, such as rehabilitation and Indigenous health, should be explored, and continuous reflection and dialogue are required to identify respectful modes of articulation that value different cultures in person-centered care that focuses on individuals and their communities.

This study is a novel contribution to the field of cultural perspectives on health and rehabilitation, aiming to incorporate the concept of equity in access to health care and the type of services provided to culturally diverse populations that have had their rights to health access violated. Regarding the limitations of this study, it does not claim to be representative, as the conditions and implementation strategies of the PESPI vary in each municipality; therefore, we suggest a situated reading of these findings. It also did not include documentary analysis that would allow for a contrast of the policy implementation and its content. New studies are needed to understand and design health care approaches that incorporate interculturality in rural and urban contexts, contributing to reducing health disparities among different peoples.
